# Performance evaluation of the binary MLC system on a novel biology‐guided radiotherapy machine using beamlet sequence log data from daily clinical treatments

**DOI:** 10.1002/acm2.70655

**Published:** 2026-06-04

**Authors:** Chunhui Han, Dave Meer, Blake Gaderlund, An Liu

**Affiliations:** ^1^ Department of Radiation Oncology City of Hope Duarte California USA; ^2^ RefleXion Medical, Inc. Hayward California USA

**Keywords:** beamlet sequence, binary MLC, image‐guided radiotherapy, quality assurance

## Abstract

**Purpose:**

To evaluate the clinical performance of a binary multi‐leaf collimator (MLC) that makes high‐frequency transitions on a novel ring‐gantry medical linac.

**Methods:**

Beamlet sequence data from treatment plans and daily treatments were retrieved for ten patients treated with image‐guided radiotherapy (IGRT) on the novel machine, with five patients treated in the pelvic and thoracic regions respectively. The prescription dose ranged from 1.8 to 7 Gy per fraction with an average beam‐on time of 484.1 ± 129.6 s (range: 299–666 s). All plans used a field size of 40 cm x 2 cm. A 64‐leaf binary MLC modulates the beam at 50 gantry firing positions in each gantry rotation while the gantry rotates at 60 RPM. Gantry angle, couch position, delivered monitor units (MU), and MLC leaf opening status were recorded at each beam firing position. Daily beamlet sequence log data was compared with those in the treatment plan at each couch position.

**Results:**

The ten treatment plans had an average MU per fraction of 5269 ± 1462 MU (range: 3184–7259) with an average total gantry firing positions of 15 835 ± 4391 (range: 9566–21 817). In daily delivery, an average number of 29.9 ± 8.9 (range: 12.3–41.0) gantry firing positions were skipped, resulting in an average reduction of 9.9 ± 3.0 MU (range: 4.1–13.6 MU), or 0.19 ± 0.04% (range: 0.13–0.28%), relative to plan MUs. The MU reduction was proportional to the number of skipped gantry firing positions, while the number of skipped gantry firing positions had an approximate linear relationship with the total number of gantry firing positions in a plan.

**Conclusion:**

In‐house software tools have been developed to efficiently evaluate daily performance of the binary MLC on the novel machine. In delivering IGRT plans, the number of skipped gantry positions or MU reduction showed no clinically significant effect on delivered dose.

## 1 INTRODUCTION

Modern radiotherapy relies heavily on the precision and reliability of multileaf collimator (MLC) systems to deliver highly conformal dose distributions while sparing surrounding healthy tissue. In image‐guided radiotherapy (IGRT), the accuracy of MLC leaf movement is particularly critical, as treatment delivery is often based on complex, high‐resolution plans involving numerous control points and rapid segment transitions. One known challenge in this context is the potential omission of low monitor unit (MU) segments, often referred to as “skipped” MUs, which can occur due to communication delays or hardware limitations between the MU console and the MLC controller.^1^ Although reports of such events are relatively rare, their potential dosimetric impact underscores the importance of evaluating MLC performance under routine clinical conditions.

**FIGURE 1:**
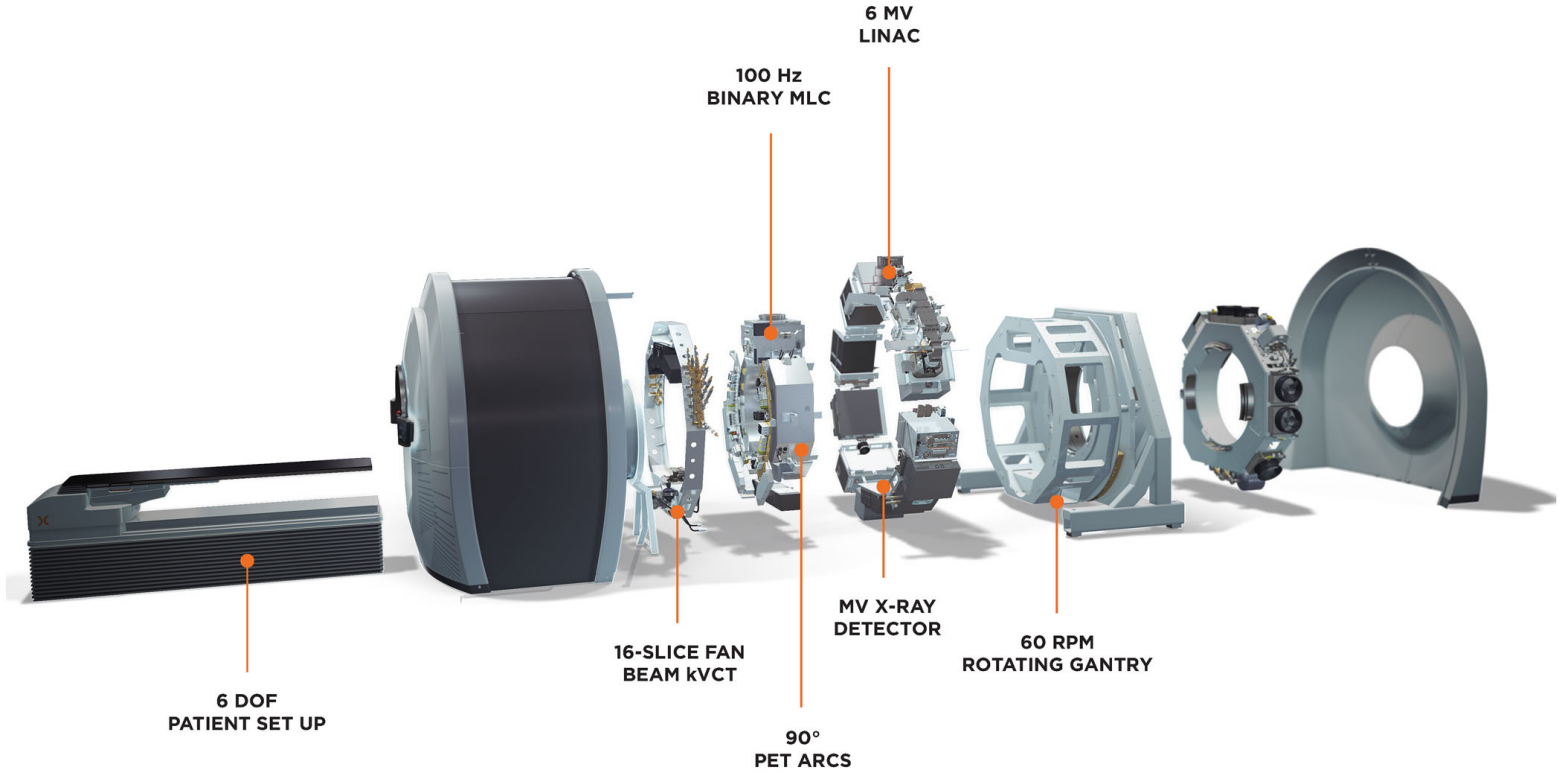
Schematic of the RefleXion X1 machine.

The RefleXion® X1 is a novel biology‐guided radiotherapy (BgRT) platform that integrates a 6‐MV flattening‐filter‐free (FFF) linac with dual 90° PET detectors, a fan‐beam kVCT imaging system, and megavoltage (MV) imaging capabilities on a high‐speed rotating ring gantry. Radiation is delivered while the gantry rotates at 60 rpm, using a 64‐leaf binary multi‐leaf collimator (MLC) system that modulates the beam at 50 firing positions per gantry rotation, and the couch advances incrementally with a step size of 2.1 mm^2^ (see Figure 1). While the X1 platform has shown promise for BgRT and IGRT workflows, no studies have been performed to evaluate performance of the MLC system in clinical treatments based on machine record. Therefore, we carried out this study to assess the clinical performance of the RefleXion X1 binary MLC during IGRT treatment courses across a range of anatomical sites and fractionation schemes. The goal is to characterize the system's real‐world reliability and inform future optimizations if needed.

## 2 METHODS AND MATERIALS

We retrospectively reviewed data from ten patients who successfully completed a radiotherapy course on the X1 machine, with five patients treated in the pelvic region and the other five in the thoracic region. Among the patients treated in the thoracic region, four were treated for locally advanced lung cancer, while the others were treated for esophageal cancer. Among the patients treated in the pelvic region, three were treated for rectal or rectosigmoid cancer, one for prostate cancer, and one for bladder cancer. Prior to the start of treatment planning, each patient received CT simulation on a CT simulator (GE Discovery 590, GE Healthcare, Chicago, IL). The CT images were sent to a treatment planning system (TPS) (Varian Eclipse version 16.1, Varian Medical Systems, Palo Alto, CA), where contours for the target volume and organs at risk (OARs) were delineated by the clinical team. Gross target volumes (GTVs) were delineated for palpable or visible disease volumes based on CT simulation images and recent diagnostic PET/CT and/or MRI images. Clinical target volumes (CTVs) were delineated to include the GTVs and potential microscopic extension of the disease. Planning target volumes (PTVs) were created by adding setup margins to the CTVs based on patient immobilization techniques used.

After contouring, the CT simulation images and structure set data were transferred to the TPS for the X1 machine for IGRT treatment plan generation. Treatment planning details for the X1 machine were described previously and are briefly present here.^3^ A flattening filter‐free 6‐MV photon beam was used to deliver radiation with a nominal dose rate of 1000 MU per minute. A pair of collimator jaws collimate the beam for a 1 or 2 cm jaw size in the superior‐inferior direction of the patient, while the maximum field width was 40 cm in the lateral direction of the patient. A 64‐leaf binary MLC was used to modulate the beam with a projected leaf width of 6.25 mm at the isocenter level. The TPS uses an accelerated proximal gradient algorithm for plan optimization, and the collapsed cone convolution superposition algorithm for final dose calculation with a dose resolution of 2.1 mm.

The average planning target volume (PTV) was 486.4 ± 500.7 cc (range: 31.1–1644.9 cc) and the average PTV length in the superior‐inferior direction was 12.2 ± 5.5 cm (range: 4.3–20.0 cm). The prescription dose ranged from 1.8 to 7 Gy per fraction with an average beam‐on time of 484.1 ± 129.6 s (range: 299–666 s) per fraction. The number of treatment fractions on the X1 ranged from 5 to 33 with an average of 19 ± 11. All treatment plans were created and delivered as IGRT plans without real‐time PET guidance. All plans used the same jaw size of 40 × 2 cm. In all plans, the treatment couch makes unidirectional movement towards the gantry (one‐pass treatment plans) without reversing couch movement direction during delivery.

During treatment delivery, the X1 gantry rotates at 60 RPM and radiation is delivered at 50 firing positions that are evenly spaced in each gantry rotation. The machine delivers a constant MU at each firing position, while the MLC aperture is modulated at each firing position. The couch remains stationary while radiation is delivered and shifts incrementally at a step size of 2.1 mm while the beam is off.

Gantry angle, couch position, delivered MU, and MLC leaf opening status at each beam firing position were recorded in daily beamlet sequence log files. These log files were retrieved for all treatment fractions. In‐house software was developed to read and parse beamlet sequence log files from daily treatments and compare the beamlet data with that in the treatment plan. Firing positions with zero MU were discarded in the analysis, while firing positions with non‐zero MU in the treatment plan but missing in daily treatment record were labeled as skipped firing positions in delivery. The following parameters were analyzed: degree of MLC leaf modulation in terms of the maximum and average leaf transition rates for each treatment plan, delivered MU in a treatment fraction compared to the plan MU, MLC leaf configuration at each firing position, and couch and firing positions in the treatment plan and daily delivery. To validate results generated by the software tools, representative parameters including total MU, MLC leaf settings, couch positions, and firing positions, were reviewed or calculated manually on the raw beamlet sequence files and compared with results from the software tools.

## 3 RESULTS

Table 1 lists relevant parameters in the treatment plan and key statistics from the beamlet sequence analysis. In all the treatment plans, the average MU per treatment fraction was 5269 ± 1462 MU (range: 3184–7259 MU) and the average total firing positions were 15 835 ± 4391 (range: 9566–21 817). For an average PTV length of 12.2 ± 5.5 cm (range: 4.3–20.0 cm), the number of couch positions in the treatment plan averaged 65.6 ± 26.4 (range: 27–103). The maximum leaf transition rate in each plan ranged from 108 to 319 leaves/sec (average: 200.0 ± 79.7 leaves/sec), while the average leaf transition rate ranged from 34.7 to 86.2 leaves/sec (average: 66.3 ± 18.3 leaves/sec).

**TABLE 1:** Parameters in the treatment plans and key statistics from beamlet sequence analysis.

Parameter	Mean ± StdDev (range)
PTV length /cm	12.2 ± 5.5 (4.3–20.0)
Dose per fraction /Gy	3.5 ± 2.1 (1.8–7.0)
Monitor Unit per fraction /MU	5,269 ± 1,462 (3184–7259)
Maximum leaf transition rate (leaves/sec)	200.0 ± 79.7 (108–319)
Average leaf transition rate (leaves/sec)	66.3 ± 18.3 (34.7–86.2)
Couch positions in the treatment plan	65.6 ± 26.4 (27–103)
Firing positions in the treatment plan	15 835 ± 4391 (9566–21 817)
Average skipped firing positions in delivery Relative to total firing positions	30.0 ± 8.9 (12.3–41.0) 0.19 ± 0.04% (0.13–0.28%)

Abbreviation: StdDev, Standard deviation.

**FIGURE 2:**
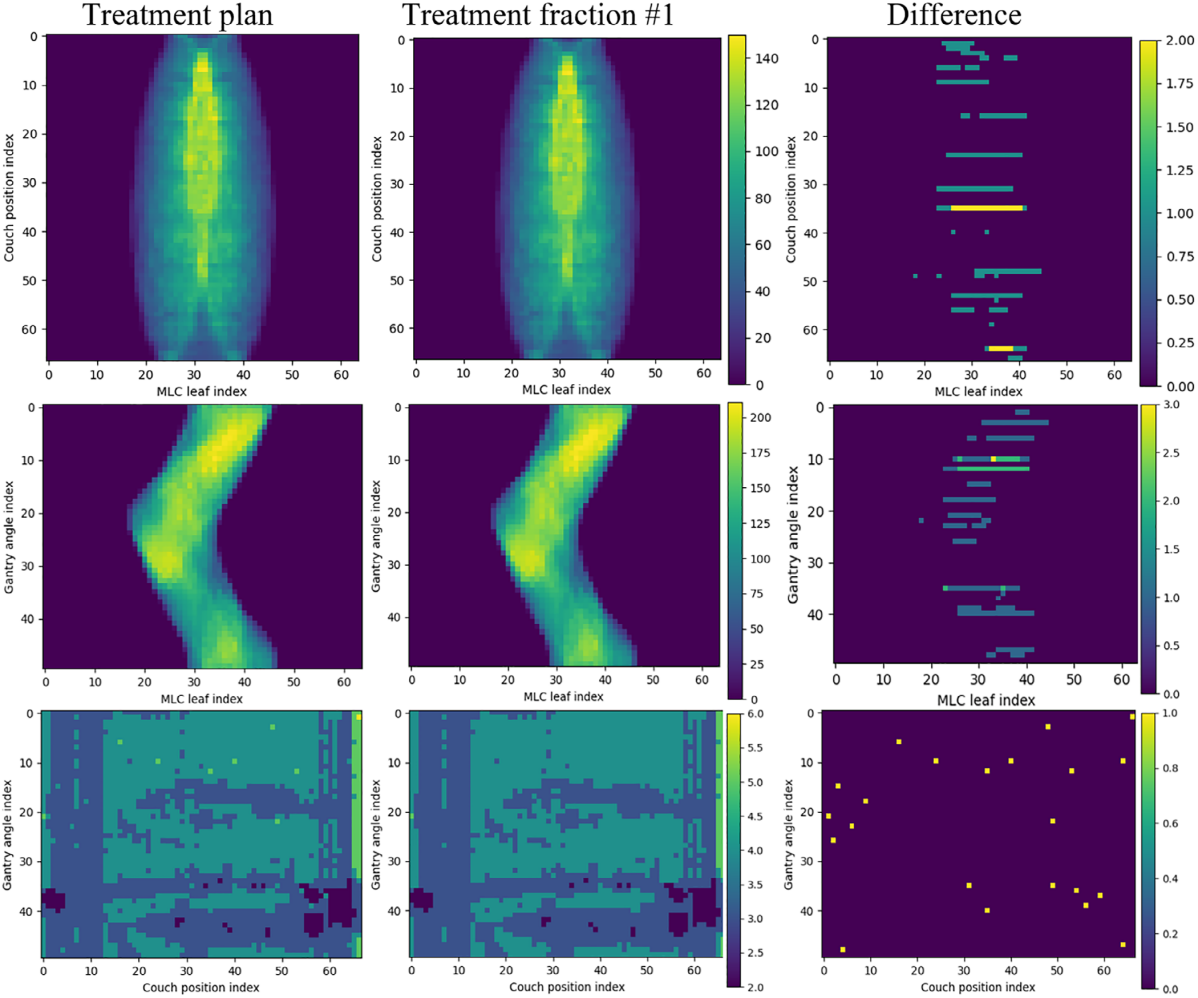
Comparison of beamlet sequence statistics between the treatment plan and the first treatment fraction for a representative patient. The first row compares number of times that an MLC leaf is open at a given couch position, summed over all gantry angles. The second row compares number of times that an MLC leaf is open at a given gantry angle, summed over all couch positions. The third row compares number of times that beam is on at a given couch position and a given gantry position. The left, middle, and right columns show data in the treatment plan, first treatment fraction, and the difference between the two, respectively.

**FIGURE 3:**
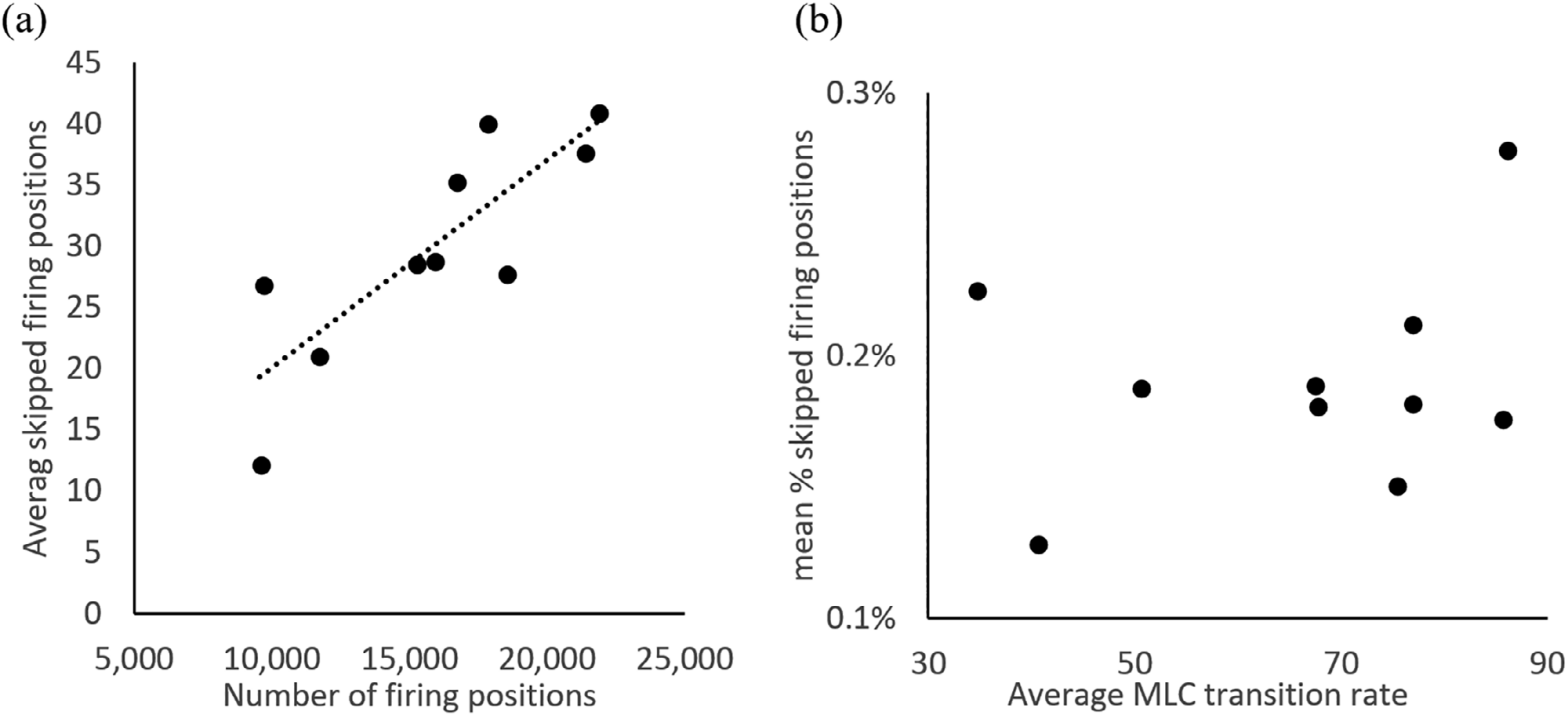
(a) Correlation between the total number of firing positions in the treatment plan and the average number of skipped firing positions in daily deliveries. (b) Correlation between the average percentage firing positions that are skipped (mean % skipped firing positions) in daily deliveries and the average MLC transition rate in the treatment plan.

Figure 2 compares beamlet sequence data in the first treatment fraction to that in the treatment plan for a representative patient in this study with 20 daily treatment fractions. Data in the treatment plan and the first treatment fraction is shown in the left and middle column, respectively, while the difference between data in the treatment plan and in the first treatment fraction is shown in the right most column. The first row of Figure 2 compares the number of times that an MLC leaf is open at a given couch position, summed over all gantry angles, in the treatment plan and the first treatment fraction. In the first treatment fraction, a maximum of 2 firing positions were skipped for any MLC leaf at a given couch position. The second row of Figure 2 compares the number of times that an MLC leaf is open at a given gantry angle, summed over all couch positions. A maximum of three firing positions were skipped for any MLC leaf at a given gantry angle. The third row of Figure 3 compares the number of times that the beam is on at a given couch position and a given gantry position. A total of 22 firing positions were skipped in the first treatment fraction compared to the treatment plan, while a total of 11 726 firing positions exists in the treatment plan. Among all 20 daily treatment fractions, the average number of skipped firing positions was 21.1 ± 2.8 (range: 11–22), or an average reduction of 0.18 ± 0.02% of total firing positions in the treatment plan. Since the same MU is delivered at each firing position, delivered MU in daily treatments was reduced by an average of 0.18 ± 0.02% (range: 0.09–0.19%).

Table 1 lists key statics from beamlet sequence analysis for all ten patients. Among all treatment plans, the average number of firing positions was 15 835 ± 4391 (range: 9566–21 817). Among all patients, the mean of average number of skipped firing positions in daily treatments was 30.0 ± 8.9 (range: 12.3–41.0). Compared to the total firing positions in the treatment plans, mean reduction in firing positions, averaged over all ten patients, was 0.19 ± 0.04% (range: 0.13–0.28%).

**FIGURE 4:**
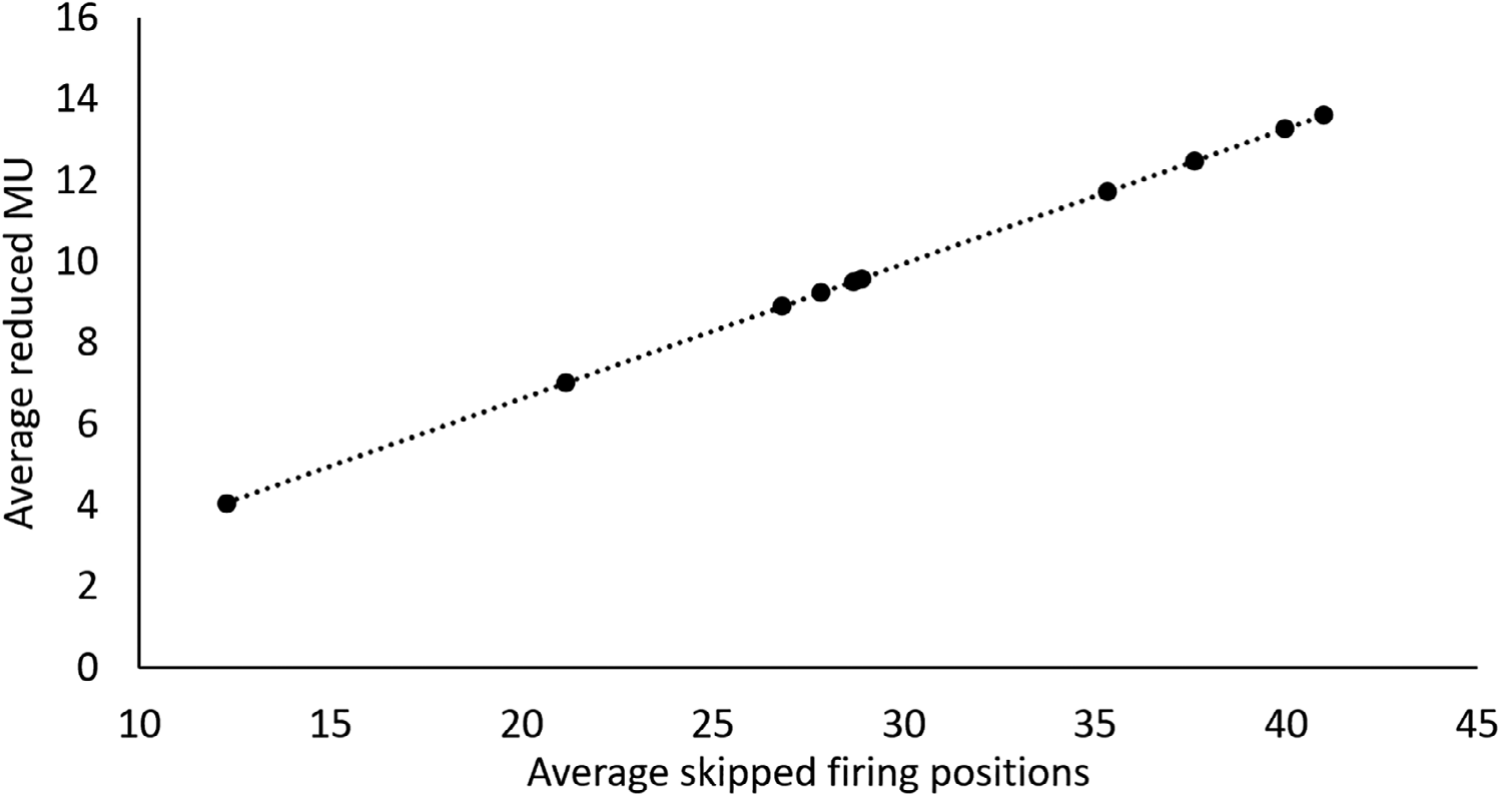
Correlation between the average number of skipped firing positions and the average reduced MU in daily deliveries for all patients in this study.

Figure 3 shows the correlation between the number of beam‐on firing positions in the treatment plan and the average number of skipped firing positions (a), and between between the average percentage skipped firing positions in daily deliveries and the average MLC transition rate in the treatment plan (b). A linear function was used to fit the data points in Figure 3a, with a R‐squared value of 0.70 and a Pearson correlation coefficient of 0.83. On the other hand, no strong correlation was found between the average percentage skipped firing positions in daily deliveries and the average MLC transition rate, with a Pearson correlation coefficient of 0.26. As a demonstration of the fact that a constant MU is delivered at any firing position, Figure 4 shows the relationship between the average number of skipped firing positions in daily deliveries and the average reduced MU in daily treatment deliveries based on daily beamlet sequence data. A linear function was used to fit the curve and the R squared value was found to be 1.000, indicating that the MU delivered is proportional to the number of firing positions in treatments.

## 4 DISCUSSION

This study is the first to assess the clinical performance of a binary multi‐leaf collimator (MLC) making high‐frequency transitions on a novel biology‐guided radiotherapy machine. This machine uses a 6‐MV linac mounted on a ring gantry with a rotation speed of 60 RPM. A 64‐leaf binary MLC modulates the beam at 50 firing positions per gantry rotation. In this single institution study, discrepancies between planned and delivered MLC modulation were clinically insignificant (Figure 2): on average, fewer than 0.3% of firing positions were skipped relative to the total number planned. Although the expected dosimetric impact is too small to be detected by conventional dosimetric measurements, the methods used in this work successfully identified skipped firing positions during daily treatments. This suggests that these techniques could be incorporated into routine QA to detect emerging machine‐performance issues before they lead to measurable dosimetric deviations, enabling proactive service interventions and minimizing potential impacts on clinical operations.

To facilitate beamlet sequence data assessment on a routine basis, in‐house software tools were developed to automatically parse, analyze, and compare beamlet sequence data from the treatment plan as well as log files from daily treatments. Figure 2 illustrates the capabilities of these tools. To enable adoption by other institutions, the software used in this study has been made publicly available.^4^ These institutions can use the code to evaluate machine performance for their own systems and integrate the analysis techniques into their routine QA workflows.

Figure 3a shows that the average number of skipped gantry firing positions in daily treatments had an approximately linear relationship with the total number of gantry firing positions in a plan. Since a constant MU is delivered per firing position, the same is true for average skipped MUs in daily deliveries compared to the plan MU per fraction. On the other hand, there was no significant correlation between the average MLC transition rate in a treatment plan and the average number of skipped gantry firing positions in daily deliveries (Figure 3b), indicating that beam modulation did not significantly affect the occurrence frequency of skipped firing positions. In fact, firing positions are skipped during delivery primarily due to transient hardware issues such as arcing in the beam generation system, low pressure in the compressed air system which holds the leaves at fully open or closed positions, and internal delays in sending system configuration information at a firing position so that the MLC cannot be configured in time. Currently, the vendor does not have performance specifications to the occurrence frequency of skipped firing positions, as the system uses skipped firing positions as a mitigation mechanism for transient hardware errors. The delivery system will issue interlocks if the hardware errors are persistent. No misfired MU was detected in this beamlet sequence data analysis, underscoring the fact that MU is only delivered at a firing position when this beam portal is properly configured.

Delivery log data can be an important tool for treatment delivery verification. Machine log analysis has been used to evaluate treatment delivery accuracy on C‐arm linacs with a two‐dimensional MLC.^5^, ^6^, ^7^ Machine log data was also used for treatment delivery verification on helical tomotherapy.^8^, ^9^, ^10^ Compared to conventional C‐arm linacs and helical tomotherapy, the gantry on the X1 machine operates with a faster rotation speed of 60 rotations per minute while the MLC leaves modulate at a higher transition frequency at 50 aperture updates per second. As the X1 is currently the only linac operating at such high gantry rotation and MLC transition frequencies, it is imperative to evaluate its performance in clinical deliveries. Beamlet sequence log data can provide valuable insight into the performance of the gantry and the MLC system during delivery.

This study has several limitations. First, results of this study were only based on the beamlet sequence data instead of direct measurements. While machine log data provides valuable information in clinical deliveries, inaccuracies or omissions may occur in the recorded log file data as demonstrated previously to other linac systems.^11^, ^12^ Verification procedures may be necessary to ensure that log file data accurately reflects actual machine positions.^13^ Second, results from this study were based on data from one machine at our institution. Machine performance, including hardware error rates, may be different on different machines. Third, this study only evaluated one‐pass IGRT plans and deliveries, As a next step, we will report beamlet sequence analysis on BGRT plans in a separate study.

## 5 CONCLUSIONS

Analysis of daily beamlet sequence log files from the RefleXion X1 system supports their use as a quality assurance approach for monitoring the accuracy of routine treatment delivery. Integration of beamlet log–based evaluation into the clinical workflow provides an effective means for ongoing surveillance of machine performance, with particular relevance to the binary multileaf collimator (MLC) system.

Evaluation of historical beamlet sequence log data from clinical IGRT treatments demonstrated that skipped firing positions and the associated monitor unit deviations from the planned delivery did not result in clinically meaningful differences in delivered dose. These findings indicate that the performance of the binary MLC system is sufficient for clinical IGRT applications.

## AUTHOR CONTRIBUTIONS

The authors contributed to the paper as follows: Study design: Chunhui Han, Dave Meer, and Blake Gaderlund. Data collection: Chunhui Han. Manuscript preparation: Chunhui Han. Manuscript review: Dave Meer, Blake Gaderlund, and An Liu. All authors reviewed the results and approved the final version of the manuscript.

## CONFLICT OF INTEREST STATEMENT

Chunhui Han and AL received research funding from RefleXion Medical, Inc. Dave Meer and Blake Gaderlund are employees of RefleXion Medical, Inc.

## Data Availability

Data in this study is stored in authors’ institutional storage space and is available upon request.
